# LIBERATE: a study protocol for midodrine for the early liberation from vasopressor support in the intensive care unit (LIBERATE): protocol for a randomized controlled trial

**DOI:** 10.1186/s13063-022-06115-0

**Published:** 2022-03-04

**Authors:** Dawn Opgenorth, Nadia Baig, Kirsten Fiest, Constantine Karvellas, Jim Kutsogiannis, Vincent Lau, Erika Macintyre, Janek Senaratne, Jocelyn Slemko, Wendy Sligl, Xiaoming Wang, Sean M. Bagshaw, Oleksa G. Rewa

**Affiliations:** 1 Department of Critical Care Medicine, Faculty of Medicine and Dentistry, University of Alberta, Edmonton, Alberta Canada; 2 Department of Critical Care Medicine, Cumming School of Medicine, University of Calgary, Calgary, Canada; 3 Alberta Health Services, Edmonton, Canada

**Keywords:** Critical care medicine, Intensive care, Randomized controlled trial, Shock; Midodrine

## Abstract

**Background:**

Intravenous (IV) vasopressors to support hemodynamics are a primary indication for intensive care unit (ICU) admission. Utilization of oral vasopressor therapy may offer an alternative to IV vasopressor therapy in the ICU, thus decreasing the need for ICU admission. Oral vasopressors, such as midodrine, have been used for hemodynamic support in non-critically ill patients, but their evaluation in critically ill patients to potentially spare IV vasopressor therapy has been limited.

**Methods:**

The LIBERATE study will be a multicenter, parallel-group, blinded, randomized placebo-controlled trial. It will recruit adult (i.e., age ≥ 18 years) critically ill patients receiving stable or decreasing doses of IV vasopressors. Eligible patients will be randomized to receive either midodrine 10 mg administered enterally every 8 h or placebo until 24 h post-discontinuation of IV vasopressors. The primary outcome will be ICU length of stay. Secondary outcomes include all-cause mortality at 90 days, hospital length of stay, length of IV vasopressor support, re-initiation of IV vasopressors, rates of ICU readmission, and occurrence of AEs. Health economic outcomes including ICU, hospital and healthcare costs, and cost-effectiveness will be evaluated. Pre-planned subgroup analyses include age, sex, frailty, severity of illness, etiology of shock, and comorbid conditions.

**Discussion:**

LIBERATE will rigorously evaluate the effect of oral midodrine on duration of ICU stay and IV vasopressor support in critically ill patients.

**Trial registration:**

ClinicalTrials.gov NCT05058612. Registered on September 28, 2021

**Supplementary Information:**

The online version contains supplementary material available at 10.1186/s13063-022-06115-0.

## Introduction

### Hemodynamic support in the ICU

Resuscitation and hemodynamic support with intravenous (IV) vasopressors is a prime indication of treatment in intensive care unit (ICU) settings [1]. Hemodynamic support is typically provided with intravenous (IV) vasopressors, catecholamines, or vasopressin [2–4]. However, the intravenous administration of vasopressors is associated with a risk of significant adverse effects including tissue necrosis, tissue thrombosis, cardiac dysrhythmias, and dysfunction as well as bowel ischemia. Furthermore, the presence of central venous catheters required for the infusion of IV vasopressors can contribute to risk of central venous catheter-associated bloodstream infections, venous thrombosis, and limited mobility [5–8]. Oral vasopressors have been historically used for hemodynamic support in non-critically ill patients, but their utility in ICU settings requires further exploration. One such oral vasopressor is midodrine (midodrine hydrochloride), which is a peripherally acting alpha-agonist that has previously been used to attenuate symptoms of chronic orthostatic hypotension, for management of intradialytic hypotension, and for treatment of hepatorenal syndrome [9–11].

### Hemodynamic support with midodrine

Previous studies have evaluated the use of midodrine for vasopressor support in the ICU (Table 1) [12–16]. The first of these studies by Levine et al. was a single-center, prospective cohort study of 20 patients which evaluated the effect of concurrent administration of midodrine on vasopressor requirements. The authors found that by using midodrine it was possible to decrease the administration rate of IV phenylephrine required for hemodynamic support [13]. Following these results, Poverno et al. evaluated the use of midodrine in 188 patients in a single-center retrospective cohort study, where the primary outcome was the duration of IV vasopressor therapy [14]. Importantly, in patients receiving midodrine, there was a significant reduction in vasopressor duration by 1.2 days. There was no change in ICU or hospital lengths of stay (LoS), nor in rates of ICU readmissions. In the recently published MIDAS study, a multicenter study conducted by Santer et al., 136 patients received oral midodrine or placebo in addition to their standard IV vasopressor therapy [15]. Study results did not find a difference in the time to vasopressor discontinuation between study groups. However, this trial was small, limited to mostly surgical patients, and was performed over 7 years. As such, the MIDAS study may have limited generalizability and may be impacted by temporal changes in critical care practice (i.e., changing co-interventions, evolving standard of care). In a larger single-center retrospective study, Whitson et al. evaluated 275 septic patients and found that midodrine use decreased IV vasopressor duration by 0.9 days (*p* < 0.001) and decreased ICU LoS by 2 days (9.4 vs. 7.5 days, *p* = 0.017) [16]. The LIBERATE study will evaluate the expanded role of midodrine for any hypotensive patient in the ICU.

**Table 1:** Previous trials evaluating the role of midodrine for hypotension

Author	Year	Patients	Population	Study type	Outcomes	Results
Levine et al.	2013	20	Surgical	Single-center prospective cohort	• **Dose of IV vasopressor**	• Decrease of −1.58 mcg/min of phenylephrine with use of midodrine
Poverno et al.	2016	188	General	Single-center retrospective cohort	• **Duration of IV vasopressor** • ICU LoS • Hospital LoS • ICU readmission	• Midodrine patients required IV vasopressors for 1.2 days following initiation of midodrine • No change in ICU length of stay • Increased hospital length of stay with midodrine (12.0 vs. 9.5days) • No difference in ICU readmission between groups
Whitson et al.	2016	275	Medical	Single-center retrospective cohort	• **Duration of IV vasopressor** • ICU LoS • Hospital LoS • IV vasopressor reinstitution • Mortality	• Decrease duration of IV vasopressors by 0.9 days • Decrease ICU length of stay (7.5 days vs. 9.4 days) with midodrine • Decrease hospital length of stay (21.9 days vs. 24.2 days) with midodrine • No mortality difference between groups
Santer et al.	2020	136	General	Multicenter RCT	• **Duration of IV vasopressor** • ICU LoS • Hospital LoS • ICU readmission • Adverse events	• No difference in duration of IV vasopressors • No difference in ICU or hospital LoS • No difference in ICU readmissions between groups

### Rationale for the study

LIBERATE hypothesizes that oral midodrine will lead to earlier liberation from IV vasopressor support in critically ill patients with refractory hypotension. Currently, literature evaluating the effects of oral midodrine on weaning from IV vasopressor support is unclear and conflicting, and the trials conducted to date have been underpowered to detect clinically important differences in patient-centered and health service endpoints [12–16]. In the environment of strained healthcare resources, limited ICU capacity, and constant pressure on ICUs worldwide, the ability to safely wean patients from IV vasopressors with transition to oral hemodynamic supporting agents may improve how patients navigate through the healthcare system [12].

## Preparatory work

Previously, we have conducted an internal pilot randomized controlled trial (RCT) (NCT04489589) that obtained Health Canada approval and No Objection Letter (obtained April 3, 2020), confirming the feasibility of patient recruitment, implementation of the protocol, and patient follow-up (Additional file 1). The experiences learned while conducting the pilot RCT informed protocol modifications in the main phase of the study as reported herein. Allocation concealment and blinding was maintained throughout this pilot, and previously recruited patients will be included in this trial.

### Primary aim and objectives

To determine the efficacy of the use of an enteral peripheral vascular vasoactive agent (i.e., midodrine hydrochloride) to facilitate liberation from continuous infusion of IV vasopressor therapy in critically ill patients with refractory hypotension.

#### Primary objectives

To compare the effect of enteral midodrine vs. placebo in critically ill patients with refractory hypotension receiving continuous IV vasopressor therapy on ICU length of stay.

#### Secondary objectives

To evaluate the effect of enteral midodrine vs. placebo on additional patient-centered outcomes in critically ill patients with refractory hypotension receiving continuous IV vasopressor therapy.

#### Tertiary objectives

To determine the health economic effects of the usage of midodrine vs. placebo in critically ill patients with refractory hypotension receiving continuous IV vasopressor therapy. The health economic analysis plan and protocol will be published separately.

## Methods

### Study design

The LIBERATE trial is a multicenter, concealed-allocation parallel-group blinded placebo-controlled randomized trial. The trial will randomly allocate 350 critically ill adult patients receiving IV vasopressor therapy to either midodrine 10 mg or identical placebo. This dose was chosen based on the product monograph suggested dosing. The investigational product will be administered enterally every 8 h for the duration of their IV vasopressor therapy and up to 24 h after discontinuation of their IV vasopressor therapy. Table 2 shows a timeline of trial activities. The SPIRIT checklist is available in Additional file 2.

**Table 2:** Trial activity timeline

Time points		Study period			
	Enrollment	Post-enrollment			
	Day 1	Days 2–30	ICU/hosp discharge	Month 6	Month 9
Enrolment					
Eligibility screen	x				
Informed consent	x				
Treatment allocation	x				
Intervention					
Midodrine or placebo	x				
Assessments					
Age, sex	x				
Weight and height	x				
Date of eligibility	x				
ICU and hospital admission date	x				
ICU admission type	x				
ICU admission diagnosis	x				
Clinical frailty scale score	x				
APACHE II score	x				
Etiology of shock	x				
Comorbid illness	x				
Vasopressor therapy	x	x			
Mechanical ventilation	x	x			
Total duration of vasopressor support			x		
Vasopressors initiated after cessation		x			
ICU or hospital death		x			
Date of ICU death or discharge		x			
ICU length of stay			x		
Hospital length of stay			x		
ICU readmission during hospital stay			x		
Discharge location from hospital			x		
Persistent organ dysfunction or death				x	x
EQ-5D					x
Adverse events	x	x	x	x	x
Cardiac events (clinically significant bradycardia, acute coronary syndrome)	x	x	x	x	x
Allergic events (paresthesia, piloerection, dysuria, pruritis, chills, pain, rash)	x	x	x	x	x
Hypertension	x	x	x	x	x
Bowel ischemia	x	x	x	x	x
Limb ischemia	x	x	x	x	x
Stroke	x	x	x	x	x
Protocol violations	x	x	x	x	x

### Study setting

The study will be centrally coordinated by the Research Office in the Department of Critical Care Medicine at the University of Alberta Hospital. The study will be performed at 7 mixed medical/surgical ICUs throughout Alberta. Additional sites outside of Alberta may be added, as necessary.

### Inclusion criteria

- Adults age ≥ 18 years receiving IV vasopressor support (i.e., norepinephrine ≥0.05 mcg/kg/min, epinephrine ≥0.05 mcg/kg/min, vasopressin ≥0.04 U/min, or phenylephrine ≥0.1mcg/kg/min) and decreasing vasopressor dose(s) (i.e., current dose less than peak dose(s))

### Exclusion criteria

- Greater than 24 h from peak vasopressor dose
- Contraindication to enteral medications or known allergy to midodrine
- Previous receipt of midodrine in last 7 days
- Expected death or anticipated withdrawal of life-sustaining therapies in next 24 h
- Pregnancy

### Rationale for eligibility criteria

The LIBERATE study is a real-world, pragmatic study for the effectiveness of utilizing midodrine, an enteral alpha-agonist, to facilitate the weaning of IV vasopressors for patients recovering from critical illness. Previously, the utility of midodrine has been studied predominantly in patients with septic shock [16]. However, as midodrine is an alpha-agonist, it can be utilized for any hypotensive condition [17]. As we are aiming to study midodrine for the facilitation of weaning of IV vasopressors, we wanted to ensure at least a stable or de-escalating dose of IV vasopressors prior to initiating enteral midodrine therapy. By excluding patients with peak doses of vasopressors > 24 h prior to randomization, we exclude a timing bias relating to the decision to initiate enteral midodrine therapy. We will be continuously assessing for maximal vasopressor dose (i.e., rolling 24-h peak vasopressor dose assessment) to ensure that any and all weaning events are identified and that we still only enroll within 24 h of peak dose.

### Recruitment strategy and approach for consent

All consecutive eligible patients will be invited to participate in the trial following referral by the treating ICU team. If the patient is unable to provide consent within the time window allowed by the protocol, his or her surrogate decision-maker (SDM) and/or legally authorized representative may be approached in person or, if necessary, contacted by telephone. The local research team could also enroll eligible patients and obtain consent subsequently as per local Research Ethics Board (REB) recommendations under a deferred consent model.

### Ethics

#### Health Canada Authorization

Since the proposed use of midodrine in the present study is outside the parameters of the Drug Identification Number (DIN) application, a Clinical Trial Application (CTA) has been submitted to Health Canada and a No Objection Letter (NOL) has been obtained (HC6-24-c236428, April 3, 2020).

#### Research ethics approval

This study was reviewed and approved by the University of Alberta Research Ethics Board (Pro00096716, August 8, 2020). All participating clinical sites will receive REB or Institutional Review Board (IRB) approval prior to commencing patient enrollment. Depending on local standards, centralized or local REBs/IRBs will approve the study protocol of each site. Before launching the trial, each clinical site will provide the coordinating center with a copy of its ethics approval letter.

#### Protocol amendments

Any modifications to the protocol which may impact the conduct of the study, potential benefit to the patient or may affect patient safety, including changes of study objectives, study design, patient population, sample sizes, study procedures, or significant administrative aspects, will require a formal amendment to the protocol. Such amendments will be reviewed and approved by the Steering Committee.

### Randomization

Randomization will be done as per a computer-generated randomization schedule through a Web-based portal (i.e., Randomize.net). Eligible patients will be randomized in a 1:1 ratio to midodrine or placebo using permuted blocks of undisclosed and variable size. We will stratify randomization by site.

Randomization will be conducted by the study pharmacists after receiving written notification of the patient enrollment from the study team. The study pharmacy will then prepare and dispense the appropriate blinded study treatment.

### Trial interventions

#### Experimental arm

Patients in the experimental arm will receive midodrine 10 mg, administered enterally, q8h from time of study inclusion to 24 h post-IV vasopressor support termination. This will be administered as a capsule containing the active substance.

#### Control arm

Patients in the control arm will receive a placebo, administered enterally, q8h from time of study inclusion to 24 h post-IV vasopressor support termination. This inactive substance will be administered as a capsule that appears identical to the midodrine capsule.

The administration of capsules will be the responsibility of the blinded bedside nurse. There is no maximum duration of study treatment.

### Rationale for interventions

#### Co-interventions

Other than the study intervention, co-interventions (i.e., steroids, IV fluids, additional vasopressors, sedation) are at the discretion of the clinical team. Co-enrollment in other studies will be permitted when that investigational product has little known hemodynamic effects and will be reviewed on a case-by-case basis.

#### Blinding

Intensivists, research personnel, ICU personnel, patients, members of the Executive and Steering Committees, and the data analysts will be blinded to the treatment allocations.

Only the pharmacist at the Research Pharmacy who is responsible for conducting the treatment allocation and preparing the study drug (i.e., midodrine or placebo) will not be blinded. In a situation needing unblinding, only the unblinded center research coordinator will have access to the treatment allocation in the Randomize.net system. Unblinding will only occur if there is deemed to be a significant safety event as per the site principal investigator.

In any case of unblinding, the data collection and follow-up schedule will be maintained.

#### Primary outcome measure

The primary outcome is:

1. The length of ICU stay as measured in hours. We will include both timing of discharge decision and actual departure from ICU.

#### Secondary outcome measures

The secondary patient-centered outcomes will include:

1. Total and post-ICU length of stay in hours
2. Duration of IV vasopressor support in hours
3. 60- and 90-day all-cause mortality
4. Rates of ICU readmission
5. Rate of re-initiation of IV vasopressors

The health economic outcomes will include:

1. ICU costs
2. Hospital costs
3. Total healthcare costs
4. Cost-effectiveness
5. Quality-adjusted life years (QALYs)

#### Safety outcome measures

It is recognized that the patient population in the ICU will experience a number of aberrations in laboratory values, signs, and symptoms due to the severity of the underlying disease and the impact of standard treatments in the ICU. These will not necessarily constitute adverse events (AEs) or serious adverse events (SAEs) unless they are considered to be related to study treatment or in the local principal investigator’s clinical judgement are not recognized events consistent with the patient’s underlying critical illness and/or chronic diseases and expected clinical course. Adverse events that will be monitored for this study are listed in Table 2.

In Canada, reporting of SAEs will follow the five recommendations for rational reporting of SAEs in investigator-initiated critical care trials of drugs in common use [18].

In contrast, unexpected safety events that are serious (i.e., fatal, life threatening, prolonging hospital stay, resulting in persistent or significant disability or incapacity, or constituting an important medical event according to local principal investigator) and considered by the local principal investigator to be at least possibly related to the trial procedures will be reported to the coordinating center within 24 h of becoming aware of the event. The coordinating center will inform applicable regulatory agencies of serious safety events possibly related to the trial procedures within 15 days of receiving the report if the safety event was neither fatal nor life-threating, or within 7 days if it was fatal or life-threatening.

Within 7 days of informing the regulatory agency of a serious unexpected safety event, the coordinating center will submit to the regulatory agency a report including an assessment of the importance and implications of findings. This information will be updated with the patient’s final status.

### Recruitment and data collection

Potentially eligible patients will be referred to the research team by intensivists and other ICU staff. The research team will confirm eligibility and the site principal investigator and co-investigators will confirm whether the patient or SDM may be approached for consent. If that is the case, the research team will obtain consent from the patient or SDM or will enroll and obtain consent as per local REB recommendations under a deferred consent model. A minimum of 4 sites in Alberta and Canada will be invited to participate with each site required to enroll at least 4 patients per month.

Daily data will be collected from day 1 until ICU discharge or 30 days, whichever occurs first. Outcome data will also be collected at 60 and 90 days. All study outcomes will be documented on the electronic case report forms (eCRF) (Additional file 3).

#### Time points

1. Baseline data: patient demographics, etiology for hypotension, severity of illness, pre-existing comorbidities, and clinical frailty scale
2. Daily data until ICU discharge or 30 days (whichever comes first): protocol adherence, co-interventions (administration of mechanical ventilation, renal replacement therapy, vasopressors, corticosteroids, intravenous fluids, blood products, sedatives, antimicrobials)
3. 60- and 90-day data: death or persistent organ dysfunction (defined as dependency on mechanical ventilation, renal replacement, or ongoing IV vasopressor use)

### Study follow-up and cohort retention

Once a patient is enrolled in the trial, the clinical site will make every reasonable effort to follow the patient for the entire duration of the study period. To minimize loss to follow-up at 90 days, the patient’s medical records will be reviewed first. If the data is not available in the medical record the patient or substitute decision-maker will be contacted directly.

Patients may withdraw their participation in the LIBERATE trial at any time. If a patient wishes to withdraw their consent from the study, we will use the following strategies to minimize the impact on the validity of the trial, meanwhile respecting the patient’s right to withdraw. We will seek a better understanding of the patient’s wishes and offer the following alternatives to complete withdrawal, which would include no further study drug exposure, data deletion, and sample destruction:

1. Discontinue study drug but allow ongoing data collection
2. Discontinue study drug and ongoing data collection but allow for initial data inclusion

Should any patients withdraw from the study, we will not seek patient replacement.

### Intention to treat and ineligible patients

We will adhere to the intention-to-treat principle and data from patients will be analyzed in the group to which they have been allocated irrespective of protocol adherence. Reasons for protocol deviations, should they arise, will be recorded. In the special case of patients mistakenly randomized, we will allow post-randomization exclusions if (1) the information about ineligibility was available at randomization, (2) two members of the Steering Committee agree that the patient was mistakenly randomized, (3) patients did not receive the intervention, and (4) patients remain blinded to their allocation. All of the above criteria must be present for patients to be excluded post-randomization [19].

Patient eligibility will be adjudicated when either a clinical site or the coordinating center suspects ineligibility. Two members of the Steering Committee will review all relevant information (i.e., hospital records) available at the time of randomization and adjudicate if the patient is eligible or ineligible. To be considered ineligible, the patient must have been ineligible at the time of randomization. If the adjudicators determine that the patient is eligible, the patient will remain in the trial. If they determine that the patient is ineligible, the patient will be withdrawn from the trial. Patients should not be withdrawn until confirmation is received from the coordinating center.

### Ancillary and post-trial care

All study subjects are critically ill and as a result will receive close monitoring as part of their standard of care prior, during, and after study participation.

### Reducing bias

Risk of selection bias will be reduced by concealed randomization using variable and undisclosed blocks. The clinical team (ICU physicians, nurses, allied health care providers), patients and family members, all research personnel, and outcome assessors and adjudicators will be blinded. Research pharmacy personnel will not be blinded as they are preparing the study drug; however, they will remain independent of the clinical team. Accordingly, decisions to discontinue life-sustaining therapies and other outcomes that require subjective assessments will not be affected by individually held beliefs regarding the effects of midodrine. In addition, we will record co-interventions to detect potential performance bias.

Finally, our biostatisticians and analysts will be blinded to allocation to ensure that blinded analysis is performed and remains in keeping with our pre-specified analytic plan.

### Substudies and secondary analyses

There are currently no substudies planned for the LIBERATE trial. However, any proposed substudies will be presented to the Executive Committee and require unanimous approval. They will then be presented to the Steering Committee and will require majority approval prior to proceeding.

### Statistical analyses

All patients will be analyzed as randomized in accordance with the intention-to-treat principle. However, we will also plan a secondary efficacy analysis including patients who received all study drug doses (per protocol analysis). The criterion for statistical significance will be based on alpha = 0.05. All tests will be two-sided, and all analysis will be performed using STATA v16 or later.

### Sample size

Using an alpha of 0.05 and a power of 80% and assuming a 20% deduction in the length of stay associated with midodrine based on previous pilot work by Anstey et al., we would need a sample size of 161 patients per arm [20]. To compensate for up to an 8% loss to follow-up, we will plan for 175 patients per arm for a total treatment size of 350 patients.

### Data analysis

#### Patient-centered analysis

All analyses will follow the intention-to-treat (ITT) principle. Analyses of the primary and secondary outcomes (i.e., patient-centered outcomes) will involve summary measures obtained by aggregating the endpoints using Stata software package (StataCorp, TX, USA). We will compare median differences along with their 95% confidence intervals. Baseline comparisons will be performed using the chi-squared test for equal proportions with results to be reported as numbers, percentages, and 95% confidence intervals. Continuous normally distributed variables will be compared using paired *t*-tests and reported as means with 95% confidence intervals, while non-normally distributed will be compared using Wilcoxon rank sum tests and reported as medians and interquartile ranges.

#### Health economic analysis

Analyses of the tertiary outcomes (i.e., health economic outcomes) will be based on health economic information obtained from public and private databases. Cost-effectiveness and cost-utility will be analyzed by estimating incremental cost and effectiveness based on ICU lengths of stay and average daily ICU costs. QALYs will be calculated based on health-related quality of life as measured by the EQ-5D-5L quality of life questionnaire. Further details of the health economic analysis will be presented separately.

### Pre-specified subgroup analyses

Secondary analyses will examine the effects for the length of ICU stay and duration of IV vasopressors within subgroups defined at baseline by age (< 65 vs. ≥65 years), sex, frailty (Clinical Frailty Scale score 1–4 vs. ≥5), severity of illness (quartiles of predicted risk of death from baseline APACHE II score), and types of shock (i.e., septic, hypovolemic, cardiogenic, obstructive or neurogenic). We will also evaluate subgroup based on comorbid conditions (i.e., pre-existing cirrhosis and acute kidney injury on admission). The subgroup analyses will use logistic regression with terms for treatment arm, subgroup, and subgroup by treatment interaction. We hypothesize that midodrine is more beneficial in young patients, in those with lower frailty and illness severity at baseline, those with pre-existing cirrhosis, those with acute kidney injury, and those who meet strict criteria for septic shock.

### Interim analysis plan

All safety events will be reviewed in real time by the PIs and the Data Safety Monitoring Committee (DSMC) chair on a case-by-case basis. They will be reported to the REB shortly after being identified. As our population is critically ill and the baseline incidence of death is high, each death will be adjudicated by the co-PIs and chair of the DSMC as related to the intervention or not.

The DSMC will review data on all possibly related AEs and SAEs after 175 patients (i.e., 50% of total planned recruitment). If the one-sided *p*-value is < 0.1 for cumulative SAE, then an interim two-sided analysis of the primary outcome will automatically be conducted. This analysis will generate a conditional power for showing efficacy in the final analysis of the primary outcome, assuming that the group-specific event rates observed to date remain the same in the total sample size. If the conditional power for efficacy is < 20%, in the context of a one-sided *p* < 0.1 for any of the safety outcomes, then the DSMC will recommend stopping the trial to the Steering Committee. The DSMC may make a similar recommendation even if these exact thresholds are not met, based on its interpretation of the balance between safety and efficacy.

### Registration

An initial ‘No Objection Letter’ was received from Health Canada on April 3, 2020 (HC6-24-c236428), and the trial was registered on www.clinicaltrials.gov on July 28, 2020 (NCT04489589). A systematic review and meta-analysis will be registered with PROSPERO (https://www.crd.york.ac.uk/prospero/).

### Data management

Clinical site research personnel will enter all patient data into the REDCap system. Briefly, the REDCap system will be managed and housed within the Department of Critical Care Medicine (DCCM) Research Office, located at the University of Alberta, Edmonton, Alberta, Canada. Personnel within DCCM Research Office will be responsible for programming and maintaining the database. With the support from the DCCM Research Office, personnel with the coordinating center will be responsible for daily data management.

### Monitoring

Multiple measures are in place for data quality control. These measures include (1) on-site training of research and clinical personnel; (2) standard operating procedures to guide storage, preparation, and administration of the study drug; (3) ongoing assessment of performance and periodic feedback to the clinical sites on performance with benchmarking from other sites; (4) site monitoring visits (remotely or in person); (5) ongoing review of missing data and outliers; and (6) rapid dissemination of responses to frequently asked questions via our study website and monthly newsletter. Coordinating center personnel and the principal investigators will be available to answer study-related questions.

### Auditing

A formal audit will be conducted by the coordinating center at regular intervals as per institutional policy. In addition, this study may be audited by other governing regulatory bodies including the University of Alberta and Health Canada.

## Trial oversight

### Executive Committee

The Executive Committee is comprised of Drs. Rewa and Bagshaw. Dr. Rewa is the principal study investigator while Dr. Bagshaw is a co-principal investigator. The committee is responsible for the day-to-day management and is accountable to the Steering Committee.

### Steering Committee

The Steering Committee consists of intensivists, investigators, and health economists and will meet quarterly by teleconference. It is comprised of Drs. Bagshaw, Fiest, Karvellas, Kutsogiannis, Lau, Macintyre, Rewa, Senaratne, Slemko, and Sligl. The Steering Committee will provide guidance and direction to the overall trial.

### Data Safety Monitoring Committee

As per the FDA guidance document the Establishment and Operation of Clinical Trial Data Monitoring Committees for Clinical Trial Sponsors, a DSMC will oversee the safety of the trial patients. The DSMC is comprised of three members who remain completely independent of the study investigators. The DSMC members all have extensive trial experience and include a senior methodologist who has served as Chair on numerous DSMCs for international RCTs, a senior biostatistician, and an intensivist. The DSMC will be responsible for safeguarding the interests of study patients, for assessing the safety and efficacy of study procedures, and for monitoring the overall conduct of the study. The DSMC will periodically review enrollment and demographic summaries, listings of protocol deviations, and summaries and listings of SAEs. In accordance with a pre-specified DSMC Charter, the DSMC will advise the Executive and Steering Committees on any concerns related to patient safety and trial conduct and will make recommendations for the study to continue as designed, for study termination, for study continuation with major or minor modifications, or temporary suspension of enrollment until some uncertainty is resolved.

## Dissemination

Dissemination will be done via an integrated knowledge translation strategy developed with input from stakeholders including care providers, patient partners, and administrators.

Plans for end-of-study dissemination include presentations at major international conferences, publications in high-impact journals and, building on experience with social media developed during previous trials, dissemination of our results via social media platforms and discussion forums managed by partner organizations.

## Discussion

Midodrine is an inexpensive and readily available intervention that may be utilized to decrease the duration of IV vasopressor therapy in refractory hypotension. This may subsequently reduce the duration and intensity for ICU support. If proven effective in decreasing ICU length of stay, midodrine could be used for any patient with refractory hypotension to liberate them from IV vasopressor therapy and facilitate earlier discharge from ICU.

The LIBERATE protocol constitutes a rigorous assessment of the effect of midodrine monotherapy on patient-centered outcomes in critically ill patients with refractory hypotension. Once completed, the LIBERATE trial is committed to harmonize data from LIBERATE and other previously published trials of enteral midodrine in an individual patient data meta-analysis and systematic review involving our study pre-determined subgroups.

## Trial status

The current protocol is version 1, last updated April 6, 2020. Patient recruitment began on 22 March 2021 and is scheduled to continue until 350 patients are recruited. The full trial schedule is listed in Additional file 4. The database will be locked after the last enrolled patient completes the 90-day follow-up, and 6 additional months will be required to address remaining data queries and to finalize the analysis.

## Supplementary Information

**Additional file 1.** Health Canada No Objection Letter

**Additional file 2.** SPIRIT Checklist

**Additional file 3.** Case Report Form

**Additional file 4.** Project Timeline

**Additional file 5.** Midodrine Product Monograph

## Data Availability

Not applicable.

## Abbreviations

AE: Adverse event
APACHE: Acute Physiology and Chronic Health Evaluation
CTA: Clinical Trials Application
DIN: Drug Identification Number
DSMC: Data Safety Monitoring Committee
eCRF: Electronic case report form
ICU: Intensive care unit
IRB: Institutional Review Board
ITT: Intention to treat
IV: Intravenous
LoS: Length of stay
NOL: No Objection Letter
QUALYs: Quality-adjusted life years
RCT: Randomized controlled trial
REB: Research Ethics Board
SAE: Serious adverse event
SDM: Substitute decision-maker
