# Supplementary material for: LIBERATE: a study protocol for midodrine for the early liberation from vasopressor support in the intensive care unit (LIBERATE): protocol for a randomized controlled trial
Source: Trials. 2022 Mar 4;23:194. doi: 10.1186/s13063-022-06115-0 (PMC8896263; doi:10.1186/s13063-022-06115-0)
Supplement: Supplementary file 3 — Additional file 3. Case Report Form [file 13063_2022_6115_MOESM3_ESM.doc]

**Midodrine for the early liberation from vasopressor support**

**LIBERATE Pilot Study**

**Principal Investigator:** Oleksa Rewa ([rewa@ualberta.ca](mailto:bagshaw@ualberta.ca))

**Co-investigator:** Sean Bagshaw ([bagshaw@ualberta.ca](mailto:rewa@ualberta.ca))

Wendy Sligl ([wsligl@ualberta.ca](mailto:wsligl@ualberta.ca))

Constantine Karvellas ([karvellas@ualberta.ca](mailto:karvellas@ualberta.ca))

**Study Coordinator:** Nadia Baig ([nadia.baig@albertahealthservices.ca](mailto:nadia.baig@albertahealthservices.ca))

|  |  |  |
| --- | --- | --- |

**AFFIX HOSPITAL PATIENT ID LABEL**

**Enrolment #:**

|  |  |  |
| --- | --- | --- |

**Patient Initials:**

| **SCREENING FOR ELIGIBILITY** |  |
| --- | --- |
| **Date of screening (dd/mm/yyyy)** | ____/____/____ |
| **INCLUSION CRITERIA (Each of criteria 1 through 3 must be fulfilled)** | |
| 1. Age ≥18 years (on the day of assessment) | [ ] Y [ ] N |
| 1. Ongoing vasopressor support (any of norepinephrine ≥0.05mcg/kg/min, epinephrine ≥0.05mcg/kg/min, vasopressin ≥0.04u/min or phenylephrine ≥0.1mcg/kg/min | [ ] Y [ ] N |
| 1. Decreasing vasopressor dose(s) (i.e., current dose less than peak dose(s)) | [ ] Y [ ] N |
| **EXCLUSION CRITERIA (Any one criterion fulfilled and the patient is ineligible)** | |
| 1. Greater than 24 hours from peak vasopressor dose | [ ] Y [ ] N |
| 1. Contraindications to enteral medications | [ ] Y [ ] N |
| 1. Previous midodrine usage in last 7 days | [ ] Y [ ] N |
| 5. Known or presumed pregnancy | [ ] Y [ ] N |
| 6. Known allergy to midodrine | [ ] Y [ ] N |
| 7. High probability of death within 24 hours or compassionate care | [ ] Y [ ] N |
| **ELIGIBILITY** | |
| According to the screening criteria above, is the patient eligible for the study? | [ ] Y [ ] N |
| *If NO → PATIENT IS EXCLUDED → skip to signature block* |  |
| **INFORMED CONSENT** | |
| Was Informed Consent obtained? | [ ] Y [ ] N |
| Was Deferred Consent obtained? | [ ] Y [ ] N |
| *If YES → Proceed to Randomization* | |

Form completed by: _____________ Signature: ____________ Date: ____/____/____

(please print name) (dd mm yyyy)

**PART 1: DEMOGRAPHICS**

| Date of birth (dd/mm/yyyy) | ____/____/________ |
| --- | --- |
| Age (yrs) |  |
| Sex: | [ ] Male [ ] Female |
| Weight: | ___________ kg |
| Height: | ___________ cm |
| Date of Study Eligibility (dd/mm/yyyy; 24hh:mm): | ____/____/________; ____/____ |
| Date of Hospital Admission (dd/mm/yyyy): | ____/____/________ |
| Date of ICU Admission (dd/mm/yyyy): | ____/____/________ |
| Type of ICU Admission | [ ] Medical [ ] Surgical |
| ICU Admission Diagnosis  CV  Respiratory  Gastrointestinal  Genitourinary/Renal  Endocrinological/Metabolic  Neurological  Trauma  Burn  Sepsis  Surgery | [ ] Y [ ] N  [ ] Y [ ] N  [ ] Y [ ] N  [ ] Y [ ] N  [ ] Y [ ] N  [ ] Y [ ] N  [ ] Y [ ] N  [ ] Y [ ] N  [ ] Y [ ] N  [ ] Y [ ] N  [ ] Y [ ] N |
| Clinical Frailty Scale Score |  |
| APACHE II Score |  |
| Etiology of Shock  Sepsis  Hypovolemia  Cardiogenic  Neurogenic  Anaphylactic  Other, specify:_________________ | [ ] Y [ ] N  [ ] Y [ ] N  [ ] Y [ ] N  [ ] Y [ ] N  [ ] Y [ ] N  [ ] Y [ ] N |

**PART 2: COMORBID ILLNESSES**

| **Co-morbid disease** |  |
| --- | --- |
| AIDS | [ ] Y [ ] N |
| Chronic Dialysis | [ ] Y [ ] N |
| Chronic Heart Failure | [ ] Y [ ] N |
| Respiratory Insufficiency | [ ] Y [ ] N |
| Cirrhosis | [ ] Y [ ] N |
| Diabetes Mellitus | [ ] Y [ ] N |
| Hepatic Failure | [ ] Y [ ] N |
| Immune Suppression | [ ] Y [ ] N |
| Leukemia | [ ] Y [ ] N |
| Lymphoma | [ ] Y [ ] N |
| Metastatic Cancer | [ ] Y [ ] N |
| Coronary Artery Disease | [ ] Y [ ] N |

**PART 3: VASOPRESSOR THERAPY:**

**Day 1 = Date of study start**

| **Day** | **Vasopressor Date**  (dd/mm/yyyy) | **Vasopressor Type** | **Minimal Daily dose** | **Maximal Daily dose** |
| --- | --- | --- | --- | --- |
| **1** |  | 1.  2.  3. |  |  |
| **2** |  | 1.  2.  3. |  |  |
| **3** |  | 1.  2.  3. |  |  |
| **4** |  | 1.  2.  3. |  |  |
| **5** |  | 1.  2.  3. |  |  |
| **6** |  | 1.  2.  3. |  |  |
| **7** |  | 1.  2.  3. |  |  |
| **8** |  | 1.  2.  3. |  |  |
| **9** |  | 1.  2.  3. |  |  |
| **10** |  | 1.  2.  3. |  |  |
| **11** |  | 1.  2.  3. |  |  |
| **12** |  | 1.  2.  3. |  |  |

| **13** |  | 1.  2.  3. |  |  |
| --- | --- | --- | --- | --- |
| **14** |  | 1.  2.  3. |  |  |
| **15** |  | 1.  2.  3. |  |  |
| **16** |  | 1.  2.  3. |  |  |
| **17** |  | 1.  2.  3. |  |  |
| **18** |  | 1.  2.  3. |  |  |
| **19** |  | 1.  2.  3. |  |  |
| **20** |  | 1.  2.  3. |  |  |
| **21** |  | 1.  2.  3. |  |  |
| **22** |  | 1.  2.  3. |  |  |
| **23** |  | 1.  2.  3. |  |  |
| **24** |  | 1.  2.  3. |  |  |
| **25** |  | 1.  2.  3. |  |  |

Add additional Vasopressor Therapy sheets as needed.

**PART 4: CO-INTERVENTIONS**

| **Day** | **Mechanical Ventilation** | **Renal Replacement Therapy** | **Corticosteroids** | **Blood Products** | **Fluid Balance** |
| --- | --- | --- | --- | --- | --- |
| **1** | [ ] Y [ ] N  Type:  [ ] Invasive  [ ] Non-Invasive  [ ] High-flow | [ ] Y [ ] N  Type:  [ ] CRRT  [ ] SLED  [ ] IHD  [ ] PD | [ ] Y [ ] N  Type:  Dose: | PRBC ________ units | + / -  _______  mls |
|  |  |  |  | Albumin  [ ] 25% [ ] 5% ______ mls |  |
|  |  |  |  | FFP ______ mls |  |
|  |  |  |  | Platelets ______ mls |  |
|  |  |  |  | Cryoprecipitate ______ mls |  |
| **2** | [ ] Y [ ] N  Type:  [ ] Invasive  [ ] Non-Invasive  [ ] High-flow | [ ] Y [ ] N  Type:  [ ] CRRT  [ ] SLED  [ ] IHD  [ ] PD | [ ] Y [ ] N  Type:  Dose: | PRBC ________ units | + / -  _______  mls |
|  |  |  |  | Albumin  [ ] 25% [ ] 5% ______ mls |  |
|  |  |  |  | FFP ______ mls |  |
|  |  |  |  | Platelets ______ mls |  |
|  |  |  |  | Cryoprecipitate ______ mls |  |
| **3** | [ ] Y [ ] N  Type:  [ ] Invasive  [ ] Non-Invasive  [ ] High-flow | [ ] Y [ ] N  Type:  [ ] CRRT  [ ] SLED  [ ] IHD  [ ] PD | [ ] Y [ ] N  Type:  Dose: | PRBC ________ units | + / -  _______  mls |
|  |  |  |  | Albumin  [ ] 25% [ ] 5% ______ mls |  |
|  |  |  |  | FFP ______ mls |  |
|  |  |  |  | Platelets ______ mls |  |
|  |  |  |  | Cryoprecipitate ______ mls |  |
| **4** | [ ] Y [ ] N  Type:  [ ] Invasive  [ ] Non-Invasive  [ ] High-flow | [ ] Y [ ] N  Type:  [ ] CRRT  [ ] SLED  [ ] IHD  [ ] PD | [ ] Y [ ] N  Type:    Dose: | PRBC ________ units | + / -  _______  mls |
|  |  |  |  | Albumin  [ ] 25% [ ] 5% ______ mls |  |
|  |  |  |  | FFP ______ mls |  |
|  |  |  |  | Platelets ______ mls |  |
|  |  |  |  | Cryoprecipitate ______ mls |  |
| **5** | [ ] Y [ ] N  Type:  [ ] Invasive  [ ] Non-Invasive  [ ] High-flow | [ ] Y [ ] N  Type:  [ ] CRRT  [ ] SLED  [ ] IHD  [ ] PD | [ ] Y [ ] N  Type:  Dose: | PRBC ________ units | + / -  _______  mls |
|  |  |  |  | Albumin  [ ] 25% [ ] 5% ______ mls |  |
|  |  |  |  | FFP ______ mls |  |
|  |  |  |  | Platelets ______ mls |  |
|  |  |  |  | Cryoprecipitate ______ mls |  |

| **Day** | **Mechanical Ventilation** | **Renal Replacement Therapy** | **Corticosteroids** | **Blood Products** | **Fluid Balance** |
| --- | --- | --- | --- | --- | --- |
| **6** | [ ] Y [ ] N  Type:  [ ] Invasive  [ ] Non-Invasive  [ ] High-flow | [ ] Y [ ] N  Type:  [ ] CRRT  [ ] SLED  [ ] IHD  [ ] PD | [ ] Y [ ] N  Type:  Dose: | PRBC ________ units | + / -  _______  mls |
|  |  |  |  | Albumin  [ ] 25% [ ] 5% ______ mls |  |
|  |  |  |  | FFP ______ mls |  |
|  |  |  |  | Platelets ______ mls |  |
|  |  |  |  | Cryoprecipitate ______ mls |  |
| **7** | [ ] Y [ ] N  Type:  [ ] Invasive  [ ] Non-Invasive  [ ] High-flow | [ ] Y [ ] N  Type:  [ ] CRRT  [ ] SLED  [ ] IHD  [ ] PD | [ ] Y [ ] N  Type:  Dose: | PRBC ________ units | + / -  _______  mls |
|  |  |  |  | Albumin  [ ] 25% [ ] 5% ______ mls |  |
|  |  |  |  | FFP ______ mls |  |
|  |  |  |  | Platelets ______ mls |  |
|  |  |  |  | Cryoprecipitate ______ mls |  |
| **8** | [ ] Y [ ] N  Type:  [ ] Invasive  [ ] Non-Invasive  [ ] High-flow | [ ] Y [ ] N  Type:  [ ] CRRT  [ ] SLED  [ ] IHD  [ ] PD | [ ] Y [ ] N  Type:  Dose: | PRBC ________ units | + / -  _______  mls |
|  |  |  |  | Albumin  [ ] 25% [ ] 5% ______ mls |  |
|  |  |  |  | FFP ______ mls |  |
|  |  |  |  | Platelets ______ mls |  |
|  |  |  |  | Cryoprecipitate ______ mls |  |
| **9** | [ ] Y [ ] N  Type:  [ ] Invasive  [ ] Non-Invasive  [ ] High-flow | [ ] Y [ ] N  Type:  [ ] CRRT  [ ] SLED  [ ] IHD  [ ] PD | [ ] Y [ ] N  Type:    Dose: | PRBC ________ units | + / -  _______  mls |
|  |  |  |  | Albumin  [ ] 25% [ ] 5% ______ mls |  |
|  |  |  |  | FFP ______ mls |  |
|  |  |  |  | Platelets ______ mls |  |
|  |  |  |  | Cryoprecipitate ______ mls |  |
| **10** | [ ] Y [ ] N  Type:  [ ] Invasive  [ ] Non-Invasive  [ ] High-flow | [ ] Y [ ] N  Type:  [ ] CRRT  [ ] SLED  [ ] IHD  [ ] PD | [ ] Y [ ] N  Type:  Dose: | PRBC ________ units | + / -  _______  mls |
|  |  |  |  | Albumin  [ ] 25% [ ] 5% ______ mls |  |
|  |  |  |  | FFP ______ mls |  |
|  |  |  |  | Platelets ______ mls |  |
|  |  |  |  | Cryoprecipitate ______ mls |  |

| **Day** | **Mechanical Ventilation** | **Renal Replacement Therapy** | **Corticosteroids** | **Blood Products** | **Fluid Balance** |
| --- | --- | --- | --- | --- | --- |
| **11** | [ ] Y [ ] N  Type:  [ ] Invasive  [ ] Non-Invasive  [ ] High-flow | [ ] Y [ ] N  Type:  [ ] CRRT  [ ] SLED  [ ] IHD  [ ] PD | [ ] Y [ ] N  Type:  Dose: | PRBC ________ units | + / -  _______  mls |
|  |  |  |  | Albumin  [ ] 25% [ ] 5% ______ mls |  |
|  |  |  |  | FFP ______ mls |  |
|  |  |  |  | Platelets ______ mls |  |
|  |  |  |  | Cryoprecipitate ______ mls |  |
| **12** | [ ] Y [ ] N  Type:  [ ] Invasive  [ ] Non-Invasive  [ ] High-flow | [ ] Y [ ] N  Type:  [ ] CRRT  [ ] SLED  [ ] IHD  [ ] PD | [ ] Y [ ] N  Type:  Dose: | PRBC ________ units | + / -  _______  mls |
|  |  |  |  | Albumin  [ ] 25% [ ] 5% ______ mls |  |
|  |  |  |  | FFP ______ mls |  |
|  |  |  |  | Platelets ______ mls |  |
|  |  |  |  | Cryoprecipitate ______ mls |  |
| **13** | [ ] Y [ ] N  Type:  [ ] Invasive  [ ] Non-Invasive  [ ] High-flow | [ ] Y [ ] N  Type:  [ ] CRRT  [ ] SLED  [ ] IHD  [ ] PD | [ ] Y [ ] N  Type:  Dose: | PRBC ________ units | + / -  _______  mls |
|  |  |  |  | Albumin  [ ] 25% [ ] 5% ______ mls |  |
|  |  |  |  | FFP ______ mls |  |
|  |  |  |  | Platelets ______ mls |  |
|  |  |  |  | Cryoprecipitate ______ mls |  |
| **14** | [ ] Y [ ] N  Type:  [ ] Invasive  [ ] Non-Invasive  [ ] High-flow | [ ] Y [ ] N  Type:  [ ] CRRT  [ ] SLED  [ ] IHD  [ ] PD | [ ] Y [ ] N  Type:    Dose: | PRBC ________ units | + / -  _______  mls |
|  |  |  |  | Albumin  [ ] 25% [ ] 5% ______ mls |  |
|  |  |  |  | FFP ______ mls |  |
|  |  |  |  | Platelets ______ mls |  |
|  |  |  |  | Cryoprecipitate ______ mls |  |
| **15** | [ ] Y [ ] N  Type:  [ ] Invasive  [ ] Non-Invasive  [ ] High-flow | [ ] Y [ ] N  Type:  [ ] CRRT  [ ] SLED  [ ] IHD  [ ] PD | [ ] Y [ ] N  Type:  Dose: | PRBC ________ units | + / -  _______  mls |
|  |  |  |  | Albumin  [ ] 25% [ ] 5% ______ mls |  |
|  |  |  |  | FFP ______ mls |  |
|  |  |  |  | Platelets ______ mls |  |
|  |  |  |  | Cryoprecipitate ______ mls |  |

| **Day** | **Mechanical Ventilation** | **Renal Replacement Therapy** | **Corticosteroids** | **Blood Products** | **Fluid Balance** |
| --- | --- | --- | --- | --- | --- |
| **16** | [ ] Y [ ] N  Type:  [ ] Invasive  [ ] Non-Invasive  [ ] High-flow | [ ] Y [ ] N  Type:  [ ] CRRT  [ ] SLED  [ ] IHD  [ ] PD | [ ] Y [ ] N  Type:  Dose: | PRBC ________ units | + / -  _______  mls |
|  |  |  |  | Albumin  [ ] 25% [ ] 5% ______ mls |  |
|  |  |  |  | FFP ______ mls |  |
|  |  |  |  | Platelets ______ mls |  |
|  |  |  |  | Cryoprecipitate ______ mls |  |
| **17** | [ ] Y [ ] N  Type:  [ ] Invasive  [ ] Non-Invasive  [ ] High-flow | [ ] Y [ ] N  Type:  [ ] CRRT  [ ] SLED  [ ] IHD  [ ] PD | [ ] Y [ ] N  Type:  Dose: | PRBC ________ units | + / -  _______  mls |
|  |  |  |  | Albumin  [ ] 25% [ ] 5% ______ mls |  |
|  |  |  |  | FFP ______ mls |  |
|  |  |  |  | Platelets ______ mls |  |
|  |  |  |  | Cryoprecipitate ______ mls |  |
| **18** | [ ] Y [ ] N  Type:  [ ] Invasive  [ ] Non-Invasive  [ ] High-flow | [ ] Y [ ] N  Type:  [ ] CRRT  [ ] SLED  [ ] IHD  [ ] PD | [ ] Y [ ] N  Type:  Dose: | PRBC ________ units | + / -  _______  mls |
|  |  |  |  | Albumin  [ ] 25% [ ] 5% ______ mls |  |
|  |  |  |  | FFP ______ mls |  |
|  |  |  |  | Platelets ______ mls |  |
|  |  |  |  | Cryoprecipitate ______ mls |  |
| **19** | [ ] Y [ ] N  Type:  [ ] Invasive  [ ] Non-Invasive  [ ] High-flow | [ ] Y [ ] N  Type:  [ ] CRRT  [ ] SLED  [ ] IHD  [ ] PD | [ ] Y [ ] N  Type:    Dose: | PRBC ________ units | + / -  _______  mls |
|  |  |  |  | Albumin  [ ] 25% [ ] 5% ______ mls |  |
|  |  |  |  | FFP ______ mls |  |
|  |  |  |  | Platelets ______ mls |  |
|  |  |  |  | Cryoprecipitate ______ mls |  |
| **20** | [ ] Y [ ] N  Type:  [ ] Invasive  [ ] Non-Invasive  [ ] High-flow | [ ] Y [ ] N  Type:  [ ] CRRT  [ ] SLED  [ ] IHD  [ ] PD | [ ] Y [ ] N  Type:  Dose: | PRBC ________ units | + / -  _______  mls |
|  |  |  |  | Albumin  [ ] 25% [ ] 5% ______ mls |  |
|  |  |  |  | FFP ______ mls |  |
|  |  |  |  | Platelets ______ mls |  |
|  |  |  |  | Cryoprecipitate ______ mls |  |

Add additional Co-intervention sheets as needed.

**PART 5: OUTCOMES**

| **1. ICU Stay** |  |
| --- | --- |
| Date/time ICU admission (dd/mm/yyyy; 24 hh:mm): | ____/____/____; ___/___ |
| Total duration of vasopressor support (hours) |  |
| Vasopressors initiated after cessation | [ ] Y [ ] N |
| Persistent Organ Dysfunction at 60 days | [ ] Y [ ] N  If yes, type : __________________________ |
| Persistent Organ Dysfunction at 90 days | [ ] Y [ ] N  If yes, type : __________________________ |
| Echocardiogram (number performed between study start and ICU discharge. Does not include bedside tests) |  |
| Death in ICU  Date of ICU Death/Discharge (dd/mm/yyyy):  ICU Length of Stay (days) | [ ] Y [ ] N  ____/____/____ |
| Death in Hospital  Date hospital death/discharge (dd/mm/yyyy):  Hospital Length of Stay (days) | [ ] Y [ ] N  ____/____/____ |
| ICU re-admission during hospital stay | [ ] Y [ ] N |
| Discharge location:  n/a  Home  Another hospital  Long-term care Facility | [ ]  [ ]  [ ]  [ ] |

**PART 6: ADVERSE EVENTS**

Were there any **adverse events** attributed to the study intervention? [ ] Y [ ] N

If YES, did they include any of the following?:

| Re-initiation of vasopressors > 24 hours post cessation | [ ] Y [ ] N |
| --- | --- |
| ICU readmission < 48 hours of discharge | [ ] Y [ ] N |
| Cardiac event(s) | [ ] Y [ ] N |
| Allergic event(s) | [ ] Y [ ] N |
| Hypertension | [ ] Y [ ] N |
| Bowel Ischemia | [ ] Y [ ] N |
| Limb Ischemia | [ ] Y [ ] N |
| Stroke | [ ] Y [ ] N |
| Other, specify: | [ ] Y [ ] N |

If YES, please complete the Adverse Event Worksheet.

**PART 7: PROTOCOL VIOLATIONS**

Were there any **protocol violations AND/OR deviations**? [ ] Y [ ] N

| Enrolled but did not meet all inclusion/exclusion criteria | [ ] Y [ ] N |
| --- | --- |
| Study medication dose missed | [ ] Y [ ] N |
| Participant accidently unblinded | [ ] Y [ ] N |
| Other, specify: | [ ] Y [ ] N |

Please provide additional details here :

**Study Completion (Attestation)**

Form completed by: _____________ Signature: ____________ Date: ____/____/____

(please print name) (dd mm yyyy)
