# Supplementary material for: LIBERATE: a study protocol for midodrine for the early liberation from vasopressor support in the intensive care unit (LIBERATE): protocol for a randomized controlled trial
Source: Trials. 2022 Mar 4;23:194. doi: 10.1186/s13063-022-06115-0 (PMC8896263; doi:10.1186/s13063-022-06115-0)
Supplement: Supplementary file 4 — Additional file 4. Project Timeline [file 13063_2022_6115_MOESM4_ESM.docx]

**LIBERATE PROJECT TIMELINE**

|  | **2020** | | | | **2021** | | | | **2022** | | | | **2023** | | | |
| --- | --- | --- | --- | --- | --- | --- | --- | --- | --- | --- | --- | --- | --- | --- | --- | --- |
| **Activity by Quarter** | **1** | **2** | **3** | **4** | **1** | **2** | **3** | **4** | **1** | **2** | **3** | **4** | **1** | **2** | **3** | **4** |
|  | Jan-Mar | Apr- Jun | Jul-Sep | Oct-Dec | Jan-Mar | Apr- Jun | Jul-Sep | Oct-Dec | Jan-Mar | Apr- Jun | Jul-Sep | Oct-Dec | Jan-Mar | Apr- Jun | Jul-Sep | Oct-Dec |
| **Pre intervention** |  |  |  |  |  |  |  |  |  |  |  |  |  |  |  |  |
| Health Canada approval |  | X |  |  |  |  |  |  |  |  |  |  |  |  |  |  |
| Ethics approval/renewal |  |  | X |  |  |  | X |  |  |  | X |  |  |  | X |  |
| CTA/Administrative approvals |  |  |  |  | X |  |  |  |  |  |  |  |  |  |  |  |
| **Intervention phase** |  |  |  |  |  |  |  |  |  |  |  |  |  |  |  |  |
| Initiate enrollment at pilot site |  |  |  |  |  | X |  |  |  |  |  |  |  |  |  |  |
| Initiate enrollment at other sites |  |  |  |  |  |  | X | X |  |  |  |  |  |  |  |  |
| Subject enrollment |  |  |  |  |  | X | X | X | X | X | X | X | X | X | X |  |
| Adverse event/SAE review |  |  |  |  |  | X | X | X | X | X | X | X | X | X | X |  |
| **Analysis** |  |  |  |  |  |  |  |  |  |  |  |  |  |  |  |  |
| Interim analysis |  |  |  |  |  |  |  |  |  | X |  |  |  |  |  |  |
| Final analysis |  |  |  |  |  |  |  |  |  |  |  |  |  |  |  | X |
| Publish manuscript |  |  |  |  |  |  |  |  |  |  |  |  |  |  |  | X |
