# Supplementary material for: LIBERATE: a study protocol for midodrine for the early liberation from vasopressor support in the intensive care unit (LIBERATE): protocol for a randomized controlled trial
Source: Trials. 2022 Mar 4;23:194. doi: 10.1186/s13063-022-06115-0 (PMC8896263; doi:10.1186/s13063-022-06115-0)
Supplement: Supplementary file 5 — Additional file 5. Midodrine Product Monograph [file 13063_2022_6115_MOESM5_ESM.pdf]

PRODUCT MONOGRAPH

PrMAR-MIDODRINE

Midodrine Hydrochloride Tablets

2.5 mg and 5 mg

Manufacturer's Standard

Vasopressor

Marcan Pharmaceuticals Inc.
77 Auriga Drive, unit#4,
Ottawa, ON, K2E 7Z7

Date of Revision:
March 15, 2018

Submission Control No: 206047

<u>**Table of Contents**</u>

PART I: HEALTH PROFESSIONAL INFORMATION 3
SUMMARY PRODUCT INFORMATION 3
INDICATIONS AND CLINICAL USE 3
CONTRAINDICATIONS 4
WARNINGS AND PRECAUTIONS 4
ADVERSE REACTIONS 6
DRUG INTERACTIONS 7
DOSAGE AND ADMINISTRATION 8
OVERDOSAGE 9
ACTION AND CLINICAL PHARMACOLOGY 9
STORAGE AND STABILITY 10
DOSAGE FORMS, COMPOSITION AND PACKAGING 11

PART II: SCIENTIFIC INFORMATION 12
PHARMACEUTICAL INFORMATION 12
CLINICAL TRIALS 14
DETAILED PHARMACOLOGY 16
TOXICOLOGY 16
REFERENCES 19

PART III: CONSUMER INFORMATION 21

^{Pr}MAR-MIDODRINE

Midodrine Hydrochloride Tablets 2.5 mg and 5 mg

PART I: HEALTH PROFESSIONAL INFORMATION

SUMMARY PRODUCT INFORMATION

Route of Administration	Dosage Form / Strength	Clinically Relevant Nonmedicinal Ingredients
Oral	Tablet / 2.5 mg, 5 mg	None<br>*For a complete listing see* ***Dosage Forms, Composition and Packaging section****.*

INDICATIONS AND CLINICAL USE

Because midodrine hydrochloride can cause marked elevation of supine blood pressure, it should only be used in patients whose lives are considerably impaired despite standard clinical care including non-pharmacologic treatment, plasma volume expansion and lifestyle alterations. The use of midodrine hydrochloride in the management of symptomatic orthostatic hypotension is based primarily on a change in a surrogate endpoint of effectiveness, an increase in systolic blood pressure measured one minute after standing, a surrogate endpoint considered likely to correspond to clinical benefits. At present, however, clinical benefits of midodrine hydrochloride, principally improved ability to carry out activities of daily life, have not been verified.

MAR-MIDODRINE (midodrine hydrochloride) may be used to attenuate symptoms of chronic orthostatic hypotension due to autonomic failure in patients with Bradbury-Eggleston syndrome, Shy-Drager syndrome, diabetes mellitus disease and Parkinson's disease.

The initiation and up-titration of midodrine therapy should be undertaken under close medical supervision in a controlled clinical setting (see **DOSAGE AND ADMINISTRATION**).

The safety of midodrine hydrochloride in patients with orthostatic hypotension due to etiology other than those named above has not been established and its use is, therefore, not recommended.

Geriatrics (>65 years of age): MAR-MIDODRINE can be used in patients 65 years or older who have adequate renal and liver function.

Pediatrics (<18 years of age): The use of MAR-MIDODRINE is not recommended in children.

CONTRAINDICATIONS

MAR-MIDODRINE (midodrine hydrochloride) is contraindicated in patients with:

- pre-existing persistent and excessive supine hypertension,
- severe organic heart disease,
- acute renal disease,
- urinary retention,
- pheochromocytoma,
- thyrotoxicosis or
- known hypersensitivity to midodrine.

WARNINGS AND PRECAUTIONS

Supine Hypertension: The most potentially serious adverse reaction associated with midodrine therapy is marked elevation of supine arterial blood pressure (supine hypertension), which, if sustained, may cause stroke, myocardial infarction, congestive heart failure, renal insufficiency or similar disorders which individually or collectively may be fatal. Symptoms of supine hypertension are more frequently detected at the initiation of midodrine therapy and during the titration period. It is essential to monitor supine and sitting blood pressures in patients maintained on midodrine.

Control of supine blood pressure has been obtained by an adjustment in midodrine dosage with or without a 45-degree elevation of the patient's head. If supine hypertension persists, treatment with midodrine should be discontinued, and appropriate therapy (e.g., phentolamine, a specific antagonist of midodrine pressor activity) instituted immediately.

Systolic hypertension of about 200 mm Hg were seen overall in about 11.8% of patients treated with 10 mg of midodrine hydrochloride, in 4 controlled clinical studies. Systolic elevation of this degree were most likely to be observed in patients with relatively elevated pre-treatment blood pressure (mean 170 mm Hg). There is no experience in patients with initial supine systolic pressure above 180 mm Hg as those patients were excluded from the clinical trials. Use of MAR-MIDODRINE in such patients is not recommended. Sitting blood pressures were also elevated by midodrine hydrochloride therapy and it is, therefore, essential to monitor supine and sitting blood pressures in patients maintained on MAR-MIDODRINE (see **DOSAGE AND ADMINISTRATION**).

To minimize the incidence of supine hypertension, instruction how to initiate midodrine therapy should strictly be followed (see **DOSAGE AND ADMINISTRATION**). Patients should be cautioned to report symptoms of supine hypertension immediately. Symptoms may include cardiac awareness, pounding in the ears, headache, blurred vision, etc. If these occur, the patient should discontinue the drug and consult with the prescribing physician.

Bradycardia: Bradycardia may occur after MAR-MIDODRINE tablets administration, primarily due to vagal reflex. Caution should be exercised when MAR-MIDODRINE is used concomitantly with cardiac glycosides (such as digitalis), psychopharmacologic agents, beta blockers or other agents which directly or indirectly reduce heart rate. Patients who experience bradycardia should be told to report immediately any signs or symptoms suggesting bradycardia (pulse slowing, increased dizziness, syncope, cardiac awareness) and to take no more drug until they have consulted with the prescribing physician.

Patients with Urinary Retention: MAR-MIDODRINE is contraindicated in patients with urinary retention.

Cardiac Arrhythmias: MAR-MIDODRINE should not be administered in the presence of uncorrected tachyarrhythmias.

General: The potential for supine and sitting hypertension should be evaluated at the beginning of midodrine therapy. Supine hypertension can often be controlled by an adjustment in midodrine dosage and/or preventing the patient from becoming fully supine, i.e., sleeping with the head of the bed elevated. The patient should be cautioned to report symptoms of supine hypertension immediately. Symptoms may include cardiac awareness, pounding in the ears, headache, blurred vision, etc. The patient should be advised to discontinue the medication immediately if supine hypertension persists.

Patients with Diabetes Mellitus: Midodrine should be used with caution in orthostatic hypotensive patients who are also diabetic, as well as those with a history of visual problems who are also taking fludrocortisone acetate, which is known to cause an increase in intraocular pressure and glaucoma.

Patients with Renal Impairment: Midodrine has not been studied in patients with renal impairment. Because desglymidodrine is eliminated via the kidneys, and higher blood levels would be expected in such patients, midodrine should be used with caution in patients with renal impairment, with a starting dose not higher than 2.5 mg (see **DOSAGE AND ADMINISTRATION**) and the patient's blood pressure should be monitored closely. Renal function should be assessed prior to initial use of midodrine and when appropriate during therapy.

Patients with Hepatic Impairment: Midodrine has not been studied in patients with hepatic impairment. Midodrine should be used with caution in patients with hepatic impairment, as the liver has a role in the metabolism of midodrine.

Urinary Retention: Midodrine may induce an increase in the tone of the internal sphincter of the urinary bladder which may lead to urinary retention. Midodrine also may effect the bladder trigone which may result in a delayed response to bladder filling. Initial signs of urinary retention are manifested clinically as hesitancy or change in frequency of micturition. Patients should be told to report promptly any indication of urinary retention (e.g., hesitancy or frequency of micturition) which may be a sign of urinary retention.

Midodrine should be used with caution in patients with urinary tract outflow obstruction, neurogenic bladder or similar conditions, since midodrine and desglymidodrine are eliminated by the kidneys and accumulation may occur in such patients.

Use in Children: Safety and effectiveness in children have not been established.

Use in Pregnancy: No teratogenic effects have been observed in studies in animals. At very high doses (20 mg/kg/day) the drug was toxic to dams and fetal loss occurred. There are no data on the use of midodrine on pregnant women. Therefore, midodrine should be used during pregnancy only when the benefit to the mother exceeds the possible harm to the fetus.

Nursing Mothers: It is not known if midodrine is excreted in human milk. Caution should be exercised when midodrine is administered to nursing mothers.

Laboratory Tests: Evaluation of the patient should include assessment of renal and hepatic function prior to initiation of therapy, and during treatment, when appropriate.

ADVERSE REACTIONS

Midodrine hydrochloride has been evaluated for safety in Phase III clinical trials in 938 patients with symptomatic orthostatic hypotension due to autonomic failure (Bradbury-Eggleston, 26.7%; Shy-Drager Syndrome, 17.8%; Parkinson's disease, 11%; diabetes mellitus, 21.2%; other conditions, 23.3%). There were 245 patients who received midodrine hydrochloride in double-blinded, placebo-controlled trials.

In the placebo-controlled studies, adverse events regardless of causality were reported in 40.4% of the patients on midodrine 10 mg t.i.d. compared to 18.6% on placebo.

In placebo-controlled trials, the rate of discontinuation of patients on midodrine 10 mg t.i.d. due to adverse events, regardless of causality, was 15.8% compared to 0% on placebo. Discontinuation due to urinary disturbance, pilomotor reactions and supine hypertension were the reasons for premature withdrawal that were more common on midodrine.

In patients treated with midodrine in which a causal relationship could not be excluded, there was one case of a serious adverse reaction reported, i.e. central retinal vein occlusion.

The most frequent (>2%) adverse events in placebo-controlled trials were supine and sitting hypertension; paraesthesia and pruritus, mainly of the scalp; goose bumps; chills; urinary urge; urinary retention and urinary frequency.

The incidence of these events in a 3-week placebo-controlled trial is shown in the following table.

	PLACEBO	MIDODRINE
**Adverse Event**	**% of patients**	**% of patients**
Paraesthesia[1]	4.5	18.3
Piloerection	0	13.4
Dsyuria[2]	0	13.4
Pruritus[3]	2.3	12.2
Supine hypertension[4]	0	7.3
Chills	0	4.9
Pain[5]	0	4.9
Rash	1.1	2.4

1 Includes paraesthesia, paraesthesia of scalp and hyperesthesia of skin.
2 Includes dysuria, increased urinary frequency, impaired urination, urinary retention (6%), urinary urgency.
3 Includes scalp pruritus (11%).
4 Includes patients with increased supine hypertension (2.4%).
5 Includes abdominal pain and pain increase.

In the 938 patients treated in controlled and uncontrolled trials, the following adverse events, regardless of causality, occurred at a frequency greater than 0.5% or occurred at a lower rate but were potentially important: asthenia, chills, dizziness, dyspepsia, dyspnea, hair disease, headache, hypertension, nausea, nervousness, pain, paraesthesia, paraesthesia scalp, piloerection, vasodilation, pruritus, pruritus scalp, rash, supine hypertension, urine retention, urine urgency.

The most potentially serious adverse reaction associated with midodrine therapy is supine hypertension. The feelings of paraesthesia, pruritus, piloerection, and chills are pilomotor reactions associated with the action of midodrine on the alpha-adrenergic receptors of the hair follicles. Feelings of urinary urgency, retention, and frequency are associated with the action of midodrine on the alpha-receptors of the bladder neck.

Laboratory Abnormalities: There were no clinical significant changes to laboratory parameters.

DRUG INTERACTIONS

Digitalis: Cardiac glycosides may enhance or precipitate bradycardia, A.V. block or arrhythmia when administered concomitantly with midodrine.

Sympathomimetic agents: The use of drugs that stimulate alpha-adrenoceptors (e.g., phenylephrine, pseudoephedrine, ephedrine, phenylpropanolamine or dihydroergotamine) may enhance or potentiate the pressor effects of midodrine. Therefore, midodrine should not be used concomitantly with vasoconstrictor sympathomimetic agents and the patient should be warned not to use over-the-counter preparations containing these drugs.

Sympatholytic agents: Alpha-adrenoceptor antagonists such as phentolamine, prazosin, doxazosin and labetalol can inhibit the vasopressor effect of midodrine.

Corticosteroids: Patients on salt-retaining steroids (e.g., fludrocortisone), with or without salt supplementation, may experience an excessive pressor effect after midodrine therapy, especially in the supine posture. The possibility of hypertensive effects with midodrine can be minimized by either reducing the dose of fludrocortisone or decreasing the salt intake prior to initiation of treatment with midodrine.

Potential for Drug Interactions: It appears possible, although there is no supporting experimental evidence, that the high renal clearance of desglymidodrine (a base) is due to active tubular secretion by the base-secreting system also responsible for the secretion of such drugs as metformin, cimetidine, ranitidine, procainamide, triamterene, flecainide, and quinidine. Thus there may be a potential for drug-drug interactions with these drugs.

DOSAGE AND ADMINISTRATION

Treatment with MAR-MIDODRINE (midodrine hydrochloride) should be started under close medical supervision in a controlled clinical setting such as in hospital, in clinic, or in the office. Hourly measurements of blood pressure (supine and sitting) should be made for 3 hours after the first dose and also the second dose of a three times daily dosage regimen. This procedure should be followed also when the dose is increased.

During the period of close medical supervision, the patient or a person living with the patient should be trained to measure blood pressure. Supine and sitting blood pressures should be measured daily for at least one month after the initiation of treatment and twice per week afterwards.

The administration of MAR-MIDODRINE should be stopped and the attending physician notified immediately, if the blood pressure in either position increases above 180/100 mm Hg.

The usual starting dose of MAR-MIDODRINE is 2.5 mg three times daily. Single doses of 2.5, 5 and 10 mg have been successfully employed. Most patients are controlled at or below 30 mg per day given in three or four divided doses. MAR-MIDODRINE can be given up to six times per day but a total daily dose of 30 mg should not be exceeded. Some patients require a morning dose that is higher than that taken later in the day. In some instances MAR-MIDODRINE has been given on a three times per day schedule as follows: 1 to 2 hours before arising in the morning, mid-morning and mid-afternoon. In order to reduce the potential for supine hypertension, it may be recommended that midodrine doses not be given after the evening meal or less than 4 hours before bedtime.

Therapy with MAR-MIDODRINE should be continued only in patients who appear to obtain symptomatic improvement during initial treatment.

The maximum recommended dose should not exceed 30 mg daily.

Dosing in children has not been studied as patients 18 years or younger were excluded from the clinical trials.

Use in the Elderly: Blood levels of midodrine and desglymidodrine were similar in patients 65 years or older versus those younger than 65 years suggesting that dosage adjustment is not necessary in elderly patients provided that their renal and liver functions are adequate.

Use in Patients with Liver and/or Renal Impairment: MAR-MIDODRINE should be used cautiously and the starting dose should not be higher than 2.5 mg (See **PRECAUTIONS** - Patients with Renal Impairment and Patients with Liver Impairment).

OVERDOSAGE

Symptoms of overdose could include hypertension, piloerection (goose bumps), a sensation of coldness and urinary retention. There are 2 reported cases of overdosage with midodrine hydrochloride, both in young males. One patient ingested midodrine hydrochloride drops, 250 mg, experienced systolic blood pressure of greater than 200 mm Hg, was treated with an IV injection of 20 mg of phentolamine, and was discharged the same night without any complaints. The other patient ingested 205 mg of midodrine hydrochloride (41 x 5 mg tablets), and was found lethargic and unable to talk, unresponsive to voice but responsive to painful stimuli, hypertensive and bradycardic. Gastric lavage was performed, and the patient recovered fully by the next day without sequelae.

The single doses that would be associated with symptoms of overdosage or would be potentially life-threatening are unknown. The oral LD_{50} is approximately 30 to 50 mg/kg in rats, 675 mg/kg in mice, and 125 to 160 mg/kg in dogs.

Recommended general treatment, based on the pharmacology of the drug, includes induced emesis and administration of alpha-sympatholytic drugs (e.g., phentolamine).

Desglymidodrine is dialyzable.

ACTION AND CLINICAL PHARMACOLOGY

Midodrine hydrochloride is a prodrug, that is, the therapeutic effect of orally administered midodrine is due to and directly related to its conversion after absorption to desglymidodrine which differs chemically from methoxamine only by lacking in a methyl group on the α carbon of the side chain.

Desglymidodrine is an α_1-adrenoceptor stimulant with little effect on cardiac β-adrenoceptors. The actions of desglymidodrine on the cardiovascular and other organ systems are essentially identical with those of other $alpha_1$-adrenoceptor stimulants, such as phenylephrine or methoxamine.

The most prominent effects of midodrine are on the cardiovascular system, consisting of a rise in standing, sitting and supine systolic and diastolic blood pressures in patients with orthostatic hypotension. Standing systolic pressure is increased by 15 to 30 mm Hg at 1 hour after a 10 mg dose of midodrine hydrochloride, with some effects persisting for another 2 to 3 hours. The increase in blood pressure is due almost entirely to an increase in peripheral resistance. Midodrine hydrochloride has no clinically significant effect on standing or supine pulse rate in patients with autonomic failure. It slightly decreases cardiac output and renal blood flow and increases the tone of the internal bladder sphincter and delays the emptying of the bladder.

Pharmacokinetics: After oral administration, midodrine is rapidly and almost completely absorbed, with a mean absolute bioavailability (as desglymidodrine) of 93%.

After the oral administration of 2.5 mg midodrine in a single dose to 12 volunteers, the mean peak concentration of unchanged midodrine is approximately 10 ng/mL and it occurs after 20-30 minutes, with a terminal plasma half-life of 0.4-0.5 hours. The mean plasma concentration of the active metabolite, desglymidodrine, peaks in approximately 1 hour, with a plasma half-life of approximately 3 hours after the oral administration of 2.5 mg midodrine.

Thorough metabolic studies have not been conducted, but it appears that deglycination of midodrine to desglymidodrine takes place in many tissues, and both compounds are metabolized in part by the liver. Neither midodrine nor desglymidodrine is a substrate for monoamine oxidase.

Both midodrine and desglymidodrine are quickly eliminated from the body, mostly by the kidneys. The renal clearance of desglymidodrine is approximately 385 mL/min, most, about 80%, by active secretion. The actual mechanism of active secretion has not been studied, but it is possible that it occurs by the base-secreting pathway responsible for the secretion of several other drugs that are bases (see **WARNINGS AND PRECAUTIONS** - Drug Interactions). Approximately 91% of the administered dose is excreted in the urine in 24 hours. Of the urinary material, 50-60% is present as desglymidodrine and approximately 2%, as non-metabolized midodrine. Unidentified breakdown products do not exceed 3.9% of the urinary material. Midodrine diffuses poorly across the blood-brain barrier.

STORAGE AND STABILITY

Store at room temperature (15-30°C).

DOSAGE FORMS, COMPOSITION AND PACKAGING

MAR-MIDODRINE (midodrine hydrochloride) tablets are available in the following strengths for oral use.

MAR-MIDODRINE Tablets 2.5 mg: Each white to off-white, round, scored tablets debossed with 'H' above the score line and 'P' below the score on one side and '504' on the other side". Available in bottles of 100.

MAR-MIDODRINE Tablets 5 mg: Each Light orange colored, round, scored tablets debossed with 'H' above the score and 'P' below the score on one side and '505' on the other side". Available in bottles of 100.

In addition to midodrine hydrochloride, each tablet contains the non-medicinal ingredients: colloidal silicon dioxide, magnesium stearate, microcrystalline cellulose and Pregelatinized starch.

MAR-MIDODRINE 5 mg tablets also contain the colourant FD&C Yellow #6 / Sunset Yellow FCF Aluminum Lake.

PART II: SCIENTIFIC INFORMATION

PHARMACEUTICAL INFORMATION

Drug Substance

Proper Name: Midodrine hydrochloride

Chemical Name:

(±)-2-Amino-*N*-(*β*-hydroxy-2,5-dimethoxyphenethyl)acetamide monohydrochloride

OR

Acetamide, 2-amino-*N*-[2-(2,5-dimethoxyphenyl)-2-hydroxy-ethyl]-, monohydrochloride, (±)-

Molecular formula and molecular weight: Base: $C_{12}H_{18}N_2O_4$, 254.29 g/mol

Salt $C_{12}H_{19}ClN_2O_4$, 290.74 g/mol

Structural Formula:

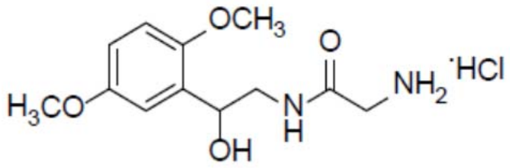

Physicochemical properties: Midodrine hydrochloride is a white crystalline powder. It is soluble in water and sparingly soluble in methanol. The following table contains the aqueous solubility of midrodrine Hydrochloride USP over the physiological pH range.

Buffer pH	Observation
1.2	Sparingly soluble
6.0	Soluble
8.0	Sparingly soluble

pKa: 7.8

pH: Between 4.0 and 5.0.

Melting range: 192.8 – 193.7°C

Partition Coefficient: -0.5

CLINICAL TRIALS

In human studies, midodrine did not affect blood sugar or urea levels in hypotensive patients or did not have any adverse effects on glucose tolerance, serum lipids, insulin or uric acid in diabetic patients. During a three-week treatment with midodrine (20 mg daily), no effects were seen on plasma clotting factor, there was no activation of fibrinolysis, or effects on number and function of thrombocytes.

Midodrine has been studied in four principal controlled trials of 4 weeks, 3 weeks, 4 days and 1 day duration, respectively. All studies were randomized, double-blind trials in patients with orthostatic hypotension of any etiology and supine-to-standing fall of systolic blood pressure of at least 15 mm Hg accompanied by at least moderate dizziness/lightheadedness. Patients with preexisting sustained supine hypertension above 180/110 mm Hg were routinely excluded. In the 4-week study in 104 patients, midodrine (10 mg) significantly elevated standing systolic blood pressure 22 mm Hg (28%) compared to 3 mm Hg (4%) for the placebo group. The 2.5 mg dose elevated blood pressure 9 mm Hg (14%). The percent increase in standing systolic pressure for all midodrine doses (2.5, 5 and 10 mg) and the 5 and 10 mg doses together was significantly superior to placebo and the dose response was linear.

In a 3-week study in 170 patients, most previously untreated with midodrine, the midodrine-treated patients (10 mg t.i.d., with the last dose not later than 6 pm) had significantly higher (by about 20 mm Hg) 1-minute standing systolic pressure 1 hour after dosing (blood pressures were not measured at other times) for all 3 weeks. After week 1, midodrine-treated patients had small improvements in dizziness/lightheadedness/unsteadiness scores and global evaluations, but these effects were made difficult to interpret by a high early drop-out rate (about 25% vs 5% on placebo). Supine and sitting blood pressure rose 16/8 and 20/10 mm Hg, respectively, on average.

In a 4-day, 4-way crossover dose-response trial, single doses of 0, 2.5, 10, and 20 mg of midodrine were given to 25 patients. The 10- and 20-mg doses produced increases in standing 1-minute systolic pressure of about 30 mm Hg at 1 hour; the increase was sustained in part for 2 hours after 10 mg and 4 hours after 20 mg. Supine systolic pressure was ≥200 mm Hg in 22% of patients on 10 mg and 45% of patients on 20 mg; elevated pressures often lasted 6 hours or more.

In a 1-day study in 70 patients, after open-label midodrine, known midodrine responders received midodrine 10 mg or placebo at 0, 3, and 6 hours. One-minute standing systolic blood pressures were increased 1 hour after each dose by about 15 mm Hg and 3 hours after each dose by about 12 mm Hg; 3-minute standing pressures were increased also at 1 but not at 3 hours after dosing. There were increases in standing time seen intermittently 1 hour after dosing, but not at 3 hours.

Comparative Bioavailability

A single centre, randomized, single oral dose, double-blind, two-treatment, two-period, two-sequence, crossover bioequivalence study comparing MAR-MIDODRINE (midodrine hydrochloride) 5 mg tablets (Marcan Pharmaceuticals Inc.) to a reference product, MIDODRINE (midodrine hydrochloride) 5 mg tablets (AA Pharma Inc.) was conducted under fasting conditions in 32 healthy male volunteers. The rate and extent of absorption of midodrine were measured and the results from the 29 subjects who completed the study are summarized in the following table:

SUMMARY TABLE OF THE COMPARATIVE BIOAVAILABILITY DATA

Midodrine<br>(1 x 5 mg)<br>From measured data<br><br>Geometric Mean<br>Arithmetic Mean (CV %)				
Parameter	Test*	Reference†	% Ratio of Geometric Means	90% Confidence Interval
AUC_T (ng*hr/mL)	20.54<br>20.86 (17.32)	20.48<br>20.74 (15.92)	100.27	97.13 - 103.52
AUC_{Inf}	20.84<br>21.16 (17.11)	20.78<br>21.03 (15.76)	100.30	97.18 - 103.53
C_{max} (ng/mL)	23.37<br>24.928 (33.810)	24.92<br>25.98 (28.19)	93.78	85.54 - 102.82
T_{max}§ (h)	0.47<br>(0.23 - 0.93)	0.47<br>(0.23 - 1.05)		
$T_{½}$€ (h)	0.50 (13.59)	0.50 (12.12)		

* MAR-MIDODRINE (midodrine hydrochloride) 5 mg tablets (Marcan Pharmaceuticals Inc.).
† MIDODRINE (midodrine hydrochloride) 5 mg tablets (AA Pharma Inc.) were purchased in Canada.
§ Expressed as the median (range) only.
€ Expressed as the arithmetic mean (CV%) only.

DETAILED PHARMACOLOGY

Preclinical Studies: Intraduodenally, midodrine was effective in rats at 2.5 to 20 mg/kg. Phentolamine (5 mg/kg i.v.), an alpha-adrenergic antagonist, inhibited the pressor response to midodrine (5 mg/kg. i.v.). Other pharmacological agents including the beta-adrenergic antagonist propranolol (5 mg/kg, i.v.) and the parasympathetic antagonist atropine (5 mg/kg, i.v.), had no influence on the pressor response.

Midodrine (1 mg/kg, i.v.) and desglymidodrine (250 µg/kg, i.v.) did not significantly affect the actions of the beta-adrenergic agonist isoprenaline (0.4 µg/kg, i.v.) on systolic or diastolic pressure, dP/dT, heart rate or femoral blood flow in the anaesthetized cat.

Desglymidodrine (10^{-6} to 10^{-4} M) caused a dose-related increase in tension of isolated femoral artery and femoral vein strips from dogs while midodrine had no effect at concentrations up to 10^{-3} M. These response curves were shifted to the right in a parallel fashion by phentolamine indicating competitive antagonism.

Neither midodrine nor desglymidodrine affected acetylcholine induced bronchospasms in guinea pigs. Neither midodrine nor desglymidodrine had any significant CNS activity in the mouse.

TOXICOLOGY

Acute Toxicity: The acute toxicity of midodrine is as follows:

	**LD_{50} mg/kg**		
	**Mouse**	**Rat**	**Dog**
Intravenous	107.7	17.69	---
Intraperitoneal	199.9	25.55	---
Subcutaneous	196.6	30.18	---
Oral	675.0	32.90	126.0 – 159.0

The toxicity pattern is identical in all 3 animal species and is characterized by exophthalmus, piloerection, dyspnea, salivation and tonoclonic spasms

Subacute Toxicity: Midodrine was given to rats (10 males and 10 females per dose) by stomach tube for up to 20 days at doses of 5, 10 and 20 mg/kg/day. Alterations of the myocardium characteristic of those produced by sympathomimetic agents, taking the form of foci of degenerative myocardial fibers and an increase in connective tissue cells, were observed and these were not clearly dose dependent. Females administered oral midodrine in doses of 5, 10 and 20 mg/kg showed fatty degeneration in the periphery of liver lobes with a decrease in glycogen content; males show cellular enlargement of the liver without fatty accumulation with cells rich in glycogen. These liver changes were dose dependent.

Chronic Toxicity: Six month toxicity studies were carried out in rats and dogs. In one experiment, Sprague-Dawley rats (10 males and 10 females per dose) were dosed orally with 1, 5 and 20 mg/kg. In a second experiment, rats (10 males and 10 females per dose) were dosed orally with 0.3 mg/kg. There was slight fatty infiltration of the liver in male rats dosed with 5 mg/kg. Changes in hepatic and plasma enzyme activities did not follow any pattern that could be related clearly to midodrine. Chronic toxicity studies for 6 months in dogs (2 males and 2 females per dose) in doses up to 12.5 mg/kg/day showed sporadic degeneration of myocardial fibers with rows of fibrocytes in the myocardium. Liver function tests remained within normal limits. Piloerection and mydriasis, initially prominent and dose dependent, gradually disappeared over several weeks of continued treatment. Vomiting occurred in the first and second week, over a period of 1-5 days. In another chronic toxicity study, dogs (3 males and 3 females per dose) were treated orally with midodrine in doses of up to 27 mg/kg/day for 6 months. The dose of 9 mg/kg/day initially produced piloerection, mydriasis and vomiting, each of which dissipated with time. At 27 mg/kg/day, mild and moderate sedation was evident and was associated with decreased food intake and weight gain. There was a significant decrease in heart and spleen weight, and a significant increase in lung, liver, and kidney weight.

Mutagenicity: No mutagenicity was seen with midodrine, according to either the Ames test, using 5 strains of *Salmonella typhimurium* (up to 1000 mg/dish), or the micronucleus test in mice (up to 50 mg/kg with *5* males and 5 females per dose).

Reproductive Studies: The effect of midodrine on reproductive function was tested by the dominant lethal assay in male CFLP mice. Oral midodrine was administered for *5* consecutive days in doses of 9, 27 and 81 mg/kg/day to male mice (20 per dose). Two days after the end of treatment, the mice were paired for one week with untreated females (120 per dose). Fetal implantation rate, litter size and post implantation loss were unaffected by treatment. Midodrine was given by gavage to Sprague-Dawley rats (20 females per dose) in dose levels of 0.1, 1.5 and 20 mg/kg/day during the sixth to the fifteenth day of pregnancy. There was no effect on pregnancy or the fetus at doses of 0.1 and 1.5 mg/kg/day. At 20 mg/kg/day, there were significant reductions in the reabsorption rate and body weight of fetuses. Dams showed significant reductions in food consumption and rate of weight gain. The effect of oral gavage of midodrine at doses of 0.1, 1.5 and 20 mg/kg/day from the sixth to the eighteenth day of pregnancy was studied in New Zealand white rabbits (10 females per dose). The progress of pregnancy and the development of the fetus was normal at the two lower doses. At a dose of 20 mg/kg/day, a post-implantation loss rate of 88.2% occurred and body weight of fetuses delivered was significantly reduced. Dams were ataxic and showed significantly reduced food intake and body weight gain.

Carcinogenicity: Long-term studies have been conducted in rats and mice at dosages of 3 to 4 times the maximum recommended daily human dose on a mg/m^2 basis, with no indication of carcinogenic effects related to midodrine.

REFERENCES

1) Bradbury S and Eggleston C Postural Hypotension: A report of three cases. American Heart Journal 1925; 1:73-86.

2) Jankovic J, Gilden JL, Hiner BC, Kaufmann H, Brown DC**,** Coghlan CH, Rubin M and Fouad-Tarazi FM: Neurogenic orthostatic hypotension: A double-blind, placebo-controlled study with midodrine. Am J Med 1993;95(1):38-48.

3) Kolassa N, Schutzenberger WG**,** Wiener H and Krivanek P: Plasma level of the prodrug midodrine and its active metabolite in comparison with the alpha-mimetic action in dogs. Arch Int Pharmacodyn Ther 1979; 238(1): 96-104.

4) Low PA, Gilden JL, Freeman R, Sheng KN and McElligott MA: Efficacy of midodrine vs placebo in neurogenic orthostatic hypotension. A randomized, double-blind multicenter study. JAMA 1997;277(13):1046-1051.

5) Pittner H, Stormann H and Enzenhofer R: Pharmacodynamic actions of midodrine, a new alpha-adrenergic stimulating agent, and its main metabolite, ST 1059. Arzneim - Forsch Drug Res 1976; 26(12):2145-2154.

6) Pittner H and Turneim K: Pulmonary and systemic circulatory effects of midodrine, a new sympathomimetic and its metabolite, ST 1059 Arch Int Pharmcodyn Ther 1974; 207(1): 180- 192.

7) Polinsky RJ, Kopin IL, Ebert EH, et al: Pharmacologic distinction of different orthostatic hypotension syndromes. Neurology 1981; 31:1-7.

8) Schirger A, Sheps SG, Thomas JE and Fealey RD: Midodrine. A new agent in the management of idiopathic orthostatic hypotension and shy-drager syndrome. Mayo Clinic Proceedings 1981; 56:429-433.

9) Shy GM and Drager GA: A neurological syndrome associated with orthostatic hypotension. Arch Neurology 1960; 2:511-527.

10) Thulesius O: Pathophysiological classification & diagnosis of orthostatic hypotension. Cardiology 1976; 61(Suppl. 1):180-190.

11) Thulesius O, Gjores JE and Berlin E: Vasoconstrictor effect of midodrine, ST 1059, noradrenaline, etilefrine and dihydroergotamine on isolated human veins. Eur J Clin Pharmacol 1979; 16:423-424.

12) Wright RA, Kaufmann HC, Perera R, Opfer-Gehrking TL, McElligott MA, Sheng KN and Low PA: A double-blind, dose-response study of midodrine in neurogenic orthostatic hypotension. Neurology 1998;51(1): 120-124.

13) Zachariah PK, Bloedow DC, Moyer TP, Sheps SG, Schirger A and Fealey RD: Pharmacodynamics of midodrine, and antihypotensive agent. Clin Pharmacal Ther 1986; 39(5):586-591.

14) Product Monograph - AMATINE® (midodrine hydrochloride) tablets. Shire Canada Inc. Date of revision: 2003-02-11.

15) Product Monograph - MIDODRINE (midodrine hydrochloride tablets 2.5 mg and 5 mg), Control No. 138681; AA Pharma Inc. Date of Preparation: May 26, 2010.

PART III: CONSUMER INFORMATION

[Pr]MAR-MIDODRINE
Midodrine Hydrochloride Tablets 2.5 mg and 5 mg

This leaflet is part III of a three-part "Product Monograph" published when MAR-MIDODRINE was approved for sale in Canada and is designed specifically for Consumers. This leaflet is a summary and will not tell you everything about MAR-MIDODRINE. Contact your doctor or pharmacist if you have any questions about the drug.

ABOUT THIS MEDICATION

What the medication is used for:
MAR-MIDODRINE is used for certain patients that have low blood pressure when in a standing position. It is used in people whose living activities are severely impaired even after other treatments are used.

What it does:
Midodrine increases blood pressure by causing blood vessels to tighten.

When it should not be used:
MAR-MIDODRINE should not be taken without first consulting your doctor if you have any of the following conditions:

- High blood pressure experienced when lying down
- Severe heart disease
- Kidney disease
- Urinary difficulties
- Pheochromocytoma (a tumour of the adrenal gland that causes excess release of hormones that regulate blood pressure)
- Overactive thyroid
- Are allergic to midodrine hydrochloride or to any of the nonmedicinal ingredients listed under "What the important nonmedicinal ingredients are"

What the medicinal ingredient is:
Midodrine hydrochloride

What the important nonmedicinal ingredients are:
MAR-MIDODRINE 2.5 mg and 5 mg tablets contains the following nonmedicinal ingredients: colloidal silicon dioxide, magnesium stearate, microcrystalline cellulose and Pregelatinized starch. MAR-MIDODRINE 5 mg tablets also contain the colourant FD&C Yellow #6 / Sunset Yellow FCF Aluminum Lake 10%.

What dosage forms it comes in:
MAR-MIDODRINE is available as 2.5 mg and 5 mg tablets.

WARNINGS AND PRECAUTIONS

Serious Warnings and Precautions
- Use of MIDODRINE can cause a marked increase in blood pressure experienced when lying down.

BEFORE you use MAR-MIDODRINE talk to your doctor or pharmacist if you:

- Have high blood pressure
- Are taking any of the medications listed in the section entitled "Interactions With This Medication"
- Have urinary difficulties
- Have an irregular heart beat
- Have low blood pressure, are diabetic or have a history of visual problems
- Have kidney or liver problems
- Are pregnant or nursing

INTERACTIONS WITH THIS MEDICATION

You should always tell your physician about all drugs you are taking or planning to take, including those obtained without a prescription.

Drugs that may interact with MAR-MIDODRINE include: digitalis glycosides (e.g. digoxin, digitoxin), adrenaline-like drugs (e.g., phenylephrine, pseudoephedrine, ephedrine, phenylpropanolamine or dihydroergotamine), alpha blockers (e.g., phentolamine, prazosin, doxazosin and labetalol and steroids that cause salt-retention (e.g. fludrocortisone).

PROPER USE OF THIS MEDICATION

Usual dose:
Treatment with MAR-MIDODRINE should be started under close medical supervision so that your blood pressure can be monitored carefully. The starting dose of MAR-MIDODRINE is 2.5 mg 3 times daily or as directed by your doctor. The drug should be taken during daytime hours, when you are likely to be upright most often. MAR-MIDODRINE should not be taken after the evening meal or less than 4 hours before bedtime.

Overdose:
Seek emergency medical attention if an overdose of this medication is suspected.

Symptoms of a midodrine overdose may include increased blood pressure (heart pounding or slower heartbeat, pounding sensation in your ears, headache or blurred vision), goose bumps, a feeling of being cold and difficulty urinating.

If you think you have taken too much MAR-MIDODRINE, contact your healthcare professional, hospital emergency department of regional poison control center immediately, even if there are no symptoms.

Missed Dose:
If a dose of this medication has been missed, it should be taken as soon as possible. However, if it is almost time for the next dose, skip the missed dose and go back to the regular dosing schedule. Do not double doses.

SIDE EFFECTS AND WHAT TO DO ABOUT THEM

The most common side effects of MAR-MIDODRINE are: High blood pressure experienced when sitting or lying down; Tingling or itching of the skin, mainly of the scalp; Goose bumps; Chills; Urinary problems; Abnormal loss of strength; Dizziness; Pain in the upper area of the stomach; Hair disease; Headache; Shortness of breath; Nausea; Nervousness; Pain; Rash

SERIOUS SIDE EFFECTS, HOW OFTEN THEY HAPPEN AND WHAT TO DO ABOUT THEM

Symptom / effect		Talk with your doctor or pharmacist		Stop taking drug and call your doctor or pharmacist
		Only if severe	In all cases	
Common	High blood pressure experienced when lying down		√	√
Uncommon	Abnormal heartbeat		√	√
Uncommon	Loss of vision (Central retinal vein occlusion)		√	√
Uncommon	Increased dizziness/faintness		√	√

This is not a complete list of side effects. For any unexpected effects while taking MAR-MIDODRINE, contact your doctor or pharmacist.

HOW TO STORE IT

Store at room temperature (15-30°C).
Store away from heat and direct light. Keep out of reach and sight of children.

REPORTING SIDE EFFECTS

You can report any suspected side effects associated with the use of health products to Health Canada by:

- **Visiting the Web page on Adverse Reaction Reporting (https://www.canada.ca/en/health-canada/services/drugs-health-products/medeffect-canada/adverse-reaction-reporting.html) for information on how to report online, by mail or by fax; or**
- **Calling toll-free at 1-866-234-2345.**

NOTE: Contact your health professional if you need information about how to manage your side effects. The Canada Vigilance Program does not provide medical advice.

MORE INFORMATION

For more information, please contact your healthcare professionals or pharmacist first, or Marcan Pharmaceuticals Inc. at: 1-855-627-2261 or visit the website at www.marcanpharma.com

The information in this document is current as of the last revision date shown below. For the most current information please visit our website or contact us directly.

This leaflet was prepared by
Marcan Pharmaceuticals Inc.
77 Auriga Drive, Unit # 4, Ottawa,
Ontario, K2E 7Z7

Last revised: March 15, 2018
