# Supplementary material for: LIBERATE: a study protocol for midodrine for the early liberation from vasopressor support in the intensive care unit (LIBERATE): protocol for a randomized controlled trial
Source: Trials. 2022 Mar 4;23:194. doi: 10.1186/s13063-022-06115-0 (PMC8896263; doi:10.1186/s13063-022-06115-0)
Supplement: Supplementary file 1 — Additional file 1. Health Canada No Objection Letter [file 13063_2022_6115_MOESM1_ESM.pdf]

Health Canada Santé Canada

Therapeutic Products Directorate
5th Floor, Holland Cross, Tower B
Address Locator # 3105A
OTTAWA, Ontario
K1A 0K9

03 April 2020

Dr. Oleska Rewa
Assistant Professor, Department of Critical Care Medicine
The Governors of the University of Alberta
2-124 Clinical Sciences Bldg, 8440-112 Street
EDMONTON, Alberta
T6G 2B7
(780) 231-3280

Your file *Votre référence*
HC6-24-c236428
Our file *Notre référence*

No Objection Letter RE: Protocol # PRO00096716 (Version 7)

Dear Dr. Rewa:

I am pleased to inform you that the information and material to support your Clinical Trial Application for **MIDODRINE**, control number **236428**, received on March 6, 2020, have been reviewed and we have no objection to your proposed study. I would remind you of the necessity of complying with the *Food and Drug Regulations,* Division 5, in the sale of this product for clinical testing. In addition, the regulations impose record keeping responsibilities on those conducting clinical trials. You are also reminded that all clinical trials should be conducted in compliance with the Therapeutic Products Directorate's *Guideline for Good Clinical Practice.*

Please note that Health Canada has implemented electronic reporting of adverse drug reactions and is currently in pilots with some sponsors. Those sponsors who have an established electronic connection with Canada Vigilance Production stream should submit their reports using the distribution rules provided to them by Health Canada, and reporting to multiple directorates is no longer required. For the sponsors who have not yet established this connection, they should continue submitting their reports to the applicable directorate by fax or by courier. The following website provides further clarification on Health Canada's adverse drug reactions reporting requirements for clinical trials:
http://www.hc-sc.gc.ca/dhp-mps/alt_formats/pdf/prodpharma/applic-demande/guide-ld/ich/efficac/e2a_pre_notice_avis-eng.pdf

Consistent with Health Canada's Notice - *Registration and Disclosure of Clinical Trial Information* of November 30, 2007, sponsors are encouraged to register their clinical trials within 21 days of the trial's onset, using a publicly available registry that conforms with international standards for registries such as: Clinicaltrials.gov (www.clinicaltrials.gov); Current Controlled Trials (www.controlled-trials.com).

Should you have any questions concerning this letter, please contact the Office of Clinical Trials (613) 941-2132.

Yours sincerely,

This document has been signed electronically using the Health Canada docuBridge system.

Larissa Lefebvre
Acting Manager, Submission Management Division
Office of Clinical Trials

LL/mw
